# High Working Memory Capacity Predicts Less Retrieval Induced Forgetting

**DOI:** 10.1371/journal.pone.0052806

**Published:** 2013-01-11

**Authors:** Jonathan T. Mall, Candice C. Morey

**Affiliations:** Department of Experimental Psychology, University of Groningen, Groningen, The Netherlands; University College London, United Kingdom

## Abstract

**Background:**

Working Memory Capacity (WMC) is thought to be related to executive control and focused memory search abilities. These two hypotheses make contrasting predictions regarding the effects of retrieval on forgetting. Executive control during memory retrieval is believed to lead to retrieval induced forgetting (RIFO) because inhibition of competing memory traces during retrieval renders them temporarily less accessible. According to this suggestion, superior executive control should increase RIFO. Alternatively, superior focused search abilities could diminish RIFO, because delimiting the search set reduces the amount of competition between traces and thus the need for inhibition. Some evidence suggests that high WMC is related to more RIFO, which is inconsistent with the focused search hypothesis.

**Methodology/Principal Findings:**

Using the RIFO paradigm, we created distinct and overlapping categories to manipulate the amount of competition between them. This overlap increased competition between some categories while exclusive use of weak exemplars ensured negligible effects of output interference and integration.

Low WMC individuals exhibited RIFO within and between overlapping categories, indicating the effect of resolving competition during retrieval. High WMC individuals only exhibited between-category RIFO, suggesting they experienced reduced competition resolution demands. Low WMC Individuals exhibited the strongest RIFO and no retrieval benefits when interference resolution demands were high.

**Conclusions/Significance:**

Our findings qualify the inhibitory explanation for RIFO by incorporating the focused search hypothesis for materials that are likely to pose extraordinary challenges at retrieval. The results highlight the importance of considering individual differences in retrieval-induced effects and qualify existing models of these effects.

## Introduction

Retrieving particular information from memory, while fundamentally important for everyday tasks, also seems to impair memory for related but unretrieved information. This phenomenon, called retrieval-induced forgetting (RIFO), might give us important insights into the way our memory system works. RIFO is believed to be caused by inhibitory processes during retrieval, which diminish accessibility of related items. RIFO has been observed in many contexts, including semantic relations [1], [2] episodic contexts [3], category recognition [4], propositional material [5] and within a foreign language acquisition context [6].

The retrieval practice paradigm has three phases. First, the study phase in which a full set of associations (e.g. Weapon - Machete) are presented and learned. Second, the participants engage in a retrieval practice phase in which some words from certain categories receive retrieval practice (RP+) while other items from the same category (RP−) and items from the remaining categories (NRP) receive no retrieval practice. Finally, in the retrieval phase memory for all associations is tested. RIFO effects are found using a variety of recall and recognition methods, including retrieval via category cues (e.g. Bird) [7], stem completion, and cue-independent tests like item recognition [8], [9] (but see [10] for criticism on these methods).

The RIFO effect is believed to be due to inhibitory executive-control processes that occur during the retrieval practice phase [11], when resolving interference of competing memory representations is necessary to retrieve the correct item. Support for the inhibition interpretation comes from the observation that unpracticed targets closely related to the practiced items seem to be less accessible after retrieval practice even when probed with a new, unstudied cue [12], [13]. Finding RIFO even with independent cues supports the notion that not only the cue-target association has been weakened, but the concept of the target itself has been temporarily inhibited. RIFO can occur even when practiced items were not successfully retrieved during the retrieval practice phase. This meant that retrieval cues, impossible to resolve, still lead to worse performance on related compared to non-practiced items [14]. The contribution of inhibition was further demonstrated in a recent study in which the necessity of resolving interference of competing memory representations was directly manipulated. Participants were exposed to orthographically similar words during a vowel counting task. Half of them were then presented with a word completion task that allowed only one of two similar words as the right answer. A later naming task revealed that the group who had to resolve interference during the word completion took longer to read the competitor words aloud [15]. Together, this evidence supports the notion that resolving interference through inhibition affects later retrieval, decreasing the likelihood of retrieving the previously inhibited concepts.

However, the inhibitory explanation for RIFO is not beyond criticism. The inhibition explanation predicts that repeated retrieval should produce additive inhibition of unrelated items, resulting in stronger RIFO, the more often an item is retrieved. Jakab and Raaijmakers [16] manipulated item strength by changing the amount of retrieval practice an item received, but found that increasing item strength did not produce stronger RIFO effects. Others have failed to replicate findings of RIFO with independent cues [12], which could mean that the memory deficit is linked to the specific retrieval cue and that blocking may be causing RIFO. Blocking occurs when the previously retrieved items are remembered in response to the retrieval cue instead of the unpracticed target items. The blocking hypothesis assumes output interference at test, but when output interference is controlled for by using recognition [8] or independent probe tests [13], [17], RIFO is still observed (but see [18] who doubt the cue-independent nature of RIFO). Lastly, populations believed to have low inhibitory executive control still exhibit RIFO; young children [19], [20], people with schizophrenia [21] and people with Alzheimer's disease [22].

It has been suggested that the absence of RIFO effects may be accounted for by factors influencing memory consolidation. For example, in some studies [2], [10] the absence of RIFO effects can be explained by integration of practiced and unpracticed items during repeated retrieval. Integrating happens when two or more items are associated with each other, which aids the retrieval of either, since items become retrieval cues for each other [23]. If integration between two targets has occurred, then practicing one target during the retrieval practice phase might still aid retrieval of the unpracticed target. Integration is more likely when participants rehearse items together, try to form meaningful interrelations [23] or when target and competitor items are strongly associated [2], [10], [24]. Stimuli designed to have few associative connections between target and competitor items tend to produce RIFO effects [1], [2], [23], [25]. Anderson and Spellman [17] explain the integration effect with a feature suppression model. While greater feature overlap may lead to more competition between items, successful retrieval strengthens shared features, offsetting the effects of inhibition. Unique features of competing items on the other hand are inhibited, decreasing the likelihood that the item will be retrieved at a later point. The feature suppression model therefore predicts RIFO when target and competitors are moderately similar, while dissimilar, non-overlapping items are not inhibited. In summary, item characteristics and their inter-relations need to be controlled for to achieve convincing RIFO effects.

More evidence against the alternative blocking explanation comes from neurological studies examining the role of prefrontal activation in RIFO. In an fMRI study, the amount of RIFO was predicted by activity during the test phase in an area associated with the retrieval of weak memories, the left anterior VLPFC [26]. Critically, no activation was found in the mid-VLPFC, commonly associated with resolving interference [27]. Activity in the mid-VLPFC would have indicated that highly activated representations block access to the related representations. Finding left anterior VLPFC activity suggests that inhibitory control processes have weakened related but unretrieved memory representations during the retrieval practice phase.

In the present study we look at executive control contributions from an individual differences perspective. Previous work indicates that executive control abilities are directly related to working memory capacity (WMC). WMC is widely believed to be not merely a measure of storage capacity [28] but also reflects the ability to control attention or suppress irrelevant information [29]–[31]. WMC has also been associated with prefrontal activation [32], [33], particularly areas related to executive control [17], [18], suggesting that the neural networks supporting executive control are more active in high WMC individuals.

With regards to executive control and RIFO, the evidence appears to be mixed. In line with the notion that executive control processes are applied during retrieval practice, RIFO disappears when a secondary task taxing executive control is introduced during retrieval practice [34]. Additionally, Bäuml and Hanslmayer [9] used operation span scores as a measure of WMC and correlated it with RIFO effects derived from an item recognition task. The positive correlation between WMC and RIFO scores suggested that high WMC individuals applied more executive inhibitory control during retrieval practice, leading to more forgetting of related items. Groome, Thorne, Grant and Pipilis [35] on the other hand found no relationship between executive control and RIFO. They tested people with a high or low capability to inhibit intrusive thoughts, an ability strongly linked with WMC [36], [37], and found no RIFO difference between groups. Together, the evidence suggests that high WMC individuals are better able to exert executive control but such a difference does not necessarily translate into stronger RIFO effects.

A possible explanation for the current discrepancies might be found when considering that inhibition comprises different sub-processes. Latent variable analysis has shown that inhibition within memory seems to be dissociable from the inhibition of response tendencies such as moving the eyes to fixate a visual target [38]. Controlled search as related to resistance to proactive inhibition thus seems unrelated to resistance to distractor interference [38]. To illustrate, in cued recall, low WMC individuals recall fewer items, make more errors, and have longer recall latencies than high WMC individuals [39]. These findings are consistent with the idea that individuals with low WMC search a bigger set of items retrieved from long term memory (LTM) than their high WMC counterparts. These differences could be explained by the specificity of retrieval cues [40]. During memory search, retrieval cues are used to discriminate between relevant and irrelevant information to reduce the amount of competition at retrieval. Unsworth and Engle [40], [41] argue that when searching for a memory trace, high WMC individuals delimit their search set by using more specific retrieval cues, while low WMC individuals use unspecific cues and thus commit more irrelevant items into their search set. Using cues less efficiently also means that performance on earlier trials should be comparable for both groups, but the accumulation of items in the search set disproportionally harms low group individuals who do not use retrieval cues as efficiently to limit entry to the search set as individuals in the high group may. This effect has been shown with the Brown Peterson task, where performance on the first trial is equal between low and high WMC but diminishes more sharply for low WMC individuals [42]. Low WMC individuals also seem to build up proactive interference faster than high WMC individuals [43], whereas release from PI is similar for both groups [44].

In this study, we aim to examine the contribution of controlled search and executive control on RIFO effects. Expanding on the findings of Bäuml and Hanslmayer [9], who show a positive relationship between WMC and RIFO, we argue that controlled search for high WMC individuals would prevent competition between items to arise and thereby diminish the need for inhibition. While Bäuml and Hanslmayer [9] used item recognition and a one-minute consolidation interval between retrieval practice and recall, we test the relationship of WMC and RIFO with the more commonly used paradigm developed by Anderson et al. [1]. To avoid output interference, we use items with low taxonomic frequencies. Items low with taxonomic frequency, or weak items, are less likely to block access to related but unpracticed items [45] but are also less likely to be falsely retrieved during retrieval practice. Since RIFO relies on competition during retrieval practice, weak items are less susceptible to RIFO [1]. To create competition while using weak exemplars, we created categories with overlapping or distinct features. For the overlapping categories (e.g. Sharp and Weapon), items shared features, (i.e. both categories contained sharp weapons) whereas distinct items did not conceivably overlap (e.g. Hobby or Cold). Each category had an equal number of items to prevent cue-overload. Although items in overlapping categories share features, they have different, specific retrieval cues (e.g., Weapon or Sharp).

We believe that Anderson and Spellman's [17] feature suppression model can be qualified by the controlled search hypothesis of Unsworth and Engle [41] in the sense that only features of items that are part of the search set are suppressed during retrieval practice. Thus, if cues are used effectively during retrieval practice, competition between items from overlapping categories (e.g. Sharp and Weapon) is less likely and RIFO effects should be small or absent. If cues are used less effectively causing items from the overlapping (but irrelevant) category to be considered in the search set, RIFO effects should be observed. High WMC individuals may use specific retrieval cues (e.g. remembering the length of a word) to limit their search to a small, appropriate set of candidates, while low WMC individuals may use unspecific retrieval cues (e.g. whether the item was a sharp weapon), resulting in a larger set of candidates to choose from, requiring more interference resolution. If high WMC individuals differ from low WMC capacity individual because of their effective use of retrieval cues, they should show little to no RIFO, unlike previous reports have suggested [9].

Our results confirmed that low WMC individuals exhibited RIFO within and between overlapping categories, suggesting that they were unable to delimit their search set effectively. High WMC individuals only exhibited between-category RIFO which suggests that they suffered less from interference. Only high WMC individuals exhibited retrieval-induced facilitation effects for overlapping items, which again indicated their ability to search long-term memory more effectively. Both findings support the focused search hypothesis, and suggest that it should continue to be incorporated into broader discussions of attentional control and memory.

## Materials and Methods

### Ethics Statement

The study was approved by the local ethics committee (“Ethische Commissie Psychologie”) and participants gave written informed consent before the study began.

### Participants

The sample consisted of 125 students from the University of Groningen (95 women, 30 men, age ranged 18–43 years, *M* = 19.88 years, *SD* = 2.65) who participated as part of their course requirements. Participants were fluent Dutch-speakers, following a university curriculum taught entirely in Dutch. Participants were tested in a room with multiple individual cubicles. The experiment was run in groups of up to 8 participants at a time. E-Prime software [46] was used to run the experiment.

### Working Memory span tasks

Participants completed computerized versions of the operation [47] and symmetry span task [48] in a prior session to determine their working memory capacity. In operation span, participants were asked to remember serially-presented consonants, interleaved with a secondary task, judging the accuracy of math equations. For each trial, different letters (F, H, J, K, L, N, P, Q, R, S, T, and Y) and equations were presented 3–7 times before participants recalled the letters in order. In symmetry span, participants were instructed to remember serially-presented locations of red squares in a 4×4 matrix interleaved with a secondary task, judging whether a block pattern was vertically symmetrical. In each trial different locations and block patterns were presented 2–5 times, before participants recalled the locations on the matrix in order. An 85% correct criterion for performance on the secondary task (math equations and symmetry judgment) was required to take part in the following experiment. Performance was measured using the count of correct trials for a maximum score of 75 for the operation Span and 42 for the symmetry span [47], [48]. Both scores were added to create a WMC composite score. Low and high groups were created using a thirtile split of the composite score with scores below 61 or above 80, respectively.

### Retrieval practice task

#### Design

Two factors were manipulated within subjects: Retrieval-Status and Set-Type. Retrieval-Status had three levels, items that received retrieval practice (RP+), related but unpracticed items (RP−) and items from categories that received no retrieval practice (NRP). Set-Type had two levels, Distinct Set (DS) and Overlap Set (OS). To ensure that Retrieval-Status was evenly distributed between the items, a random selection of three items per category was associated equally often with each Retrieval-Status. Counterbalancing of items resulted in eight different lists, which were randomly assigned to participants.

#### Word stimuli

Ten categories from Dutch category norms [unpublished data, see appendix] were selected. Eight categories (food, cold, hobby, soft, sharp, weapon, flying, animal) were used as experimental categories and two categories (loud, swim) as fillers. Two pairs of related categories (sharp, weapons and flying, animals) formed the overlap set (OS) and the four remaining experimental categories formed the distinct set (DS). Distinct set categories were created with words that could not be confused as being members of another category, (e.g. words like “ice cream” that could fit into Food or Cold and were excluded). The category names were unambiguous, single words, with lengths between 3 and 6 letters. Words had a low average taxonomic frequency (*M±SD* = 62±31.38, Median = 60.5, range = 16–136). Items were chosen with a length between three and eight letters (*M±SD* = 5.06±1.28), and had between one and three syllables. No two items within a category or between the related categories began with the same initial letter. See Appendix, for the complete word list.

#### Study lists

For each study list, 12 filler and 48 experimental category-item pairs were constructed. Similarly to previous experiments (e.g. [1]), six experimental blocks were created to ensure that items assigned to various retrieval statuses were fairly dispersed across the study period. In each block, one item was randomly selected from each of the eight categories. To ensure even presentation of eventual RP+ and RP− items, the first block featured an RP+ item from one half of the to-be-practiced categories and an RP− item from the other half (see [49]). Subsequent blocks presented RP+ and RP− items in an alternating order. Study lists began with the two filler categories. The study lists were presented once.

#### Retrieval-practice lists

Category-target associations were practiced by retrieving a specific item given a category-plus-one-letter-stem. The practiced items, three per category, came from two of the distinct sets, two of the overlap sets and from each of the two filler categories. Each category-item pair was practiced one time resulting in 18 exemplars per list. To maximize the impact of retrieval practice, RP+ items were presented in an expanding schedule with interleaved tests of filler items, ordered to produce an expanding sequence of inter-test intervals (see [1]). There were on average 4.7 items presented between two exemplars from the same category. No two category members were presented adjacently.

#### Test lists

In the test list a category name and the initial letter of the tested item was provided. Cued recall began with a filler category followed by the eight experimental categories. Half of the experimental categories began with a practiced category and the other half with a non-practiced category. Practiced and unpractised categories were subsequently presented in an alternating order. Within a list, half of the practiced categories began with randomly selected RP+ items, the other half began with randomly selected non-practiced RP− items. In total, 54 category-item pairs were tested; the second filler category was not tested.

### Procedure

The procedure followed the retrieval practice paradigm developed by Anderson et al. [1]. The experiment consisted of five phases: Study, retrieval practice, filler task, cued recall and the free recall. In the study phase, participants were instructed to study category-exemplar combinations and to remember the exemplars by relating them to their category. Each trial consisted of a central fixation point for 1000 ms, followed by a blank screen for 500 ms, followed by one of the “category – exemplar” combinations for 5 s, followed by another blank screen for 500 ms, before the next trial began.

In the retrieval practice phase, participants were instructed to complete category-plus-one-letter-stem cues for the RP+ and filler items, with exemplars that were learned during the study phase. A trial began with a fixation point for 1000 ms, followed by a blank screen for 500 ms, followed by a category-plus-one-letter stem cue (e.g. Hobby – R_____) with an empty square underneath. Participants entered their response and after they confirmed by pressing Enter, the correct answer was shown for 2 s (e.g. Hobby – Rugby), followed by another blank screen for 500 ms, before the next trial began.

Next participants completed a filler task, a 25-minute visual change detection task. This was meant to allow time for consolidation of the category-exemplar pairs into long-term memory, while preventing active rehearsal of these materials. In the cued recall phase, participants were instructed to complete category-plus-one-letter-stem cues of all items, with exemplars learned during the study phase. Each trial began with a fixation point for 1000 ms, followed by a blank screen for 500 ms, and finally a category-plus-one-letter-stem cue with an empty square underneath. Participants were asked to respond within 7s and press enter to get to the next cue or press enter immediately to indicate that they do not know the correct answer. The whole experimental session lasted about 60 minutes.

## Results

All statistical analyses employed two-tailed tests. Post-hoc tests were Bonferroni-corrected and an alpha level of .05 was used throughout the analysis.

### Retrieval Practice Phase

For the first retrieval phase, the percentage correct recall for RP+ items from the two Set-Types was calculated per subject. We used a two (Set-Type) by three (WMC Bin) repeated measures analysis of variance (ANOVA) with a Greenhouse-Geisser correction. Set-Type (OS RP+, DS RP+) was entered as a within-subject factor and WMC bin (1–3) as a between-subject factor. We found that recall was reliably higher for distinct (*M* = 45.5%) than for overlap items (*M* = 34.1%), *F*(1,122) = 22.23, *MSE* = .81, *η^2^_p_* = .15, *p*<.001. A reliable interaction was found between Set-Type and WMC, *F*(2,122) = 6.67, *MSE* = .24, *η^2^_p_* = .10, *p* = .002. Post-hoc comparisons indicated that for lowest WMC individuals, retrieval of overlapping RP+ items was worse than for distinct RP+ items (*M* = 28.6% and 51.2%, *p*<.0001) whereas for highest WMC individuals, retrieval was comparable (*M* = 44.0% and 43.2%, *p* = .84). No other effects or interactions were found (*p*s = .10–.62).

Retrieval success rate was lower than the 74% success rate reported for weak category exemplars in previous research [1]. This difference was expected and can be accounted for by the use of category-plus-one-letter-stem cues instead of category-plus-two-letters-stem, the single presentation during retrieval practice and the items' low taxonomic frequency (rank order *M* = 62 compared to *M* = 33 according to [50] in [1]). In summary, while overall retrieval success was moderate, low WMC individuals showed worse retrieval of overlapping RP+ items than high WMC individuals.

### Reaction times during retrieval practice

A repeated measures ANOVA with Set-Type as the within-subjects variable and WMC as the between-subjects variable yielded no reliable effect or interaction (*p*s = .45–.79) on mean response times, providing no evidence that speed of successful retrieval during practice differed between groups or Set-Types.

### Cued Recall Test

To investigate the effects of Retrieval-Status and Set-Type on cued recall, recall rates were computed for RP+, RP− and NRP items within the distinct and overlap set in all lists. A repeated measures ANOVA was conducted with Retrieval-Status (RP+, RP− and NRP) and Set-Type as within-subjects factors and WMC (thirtiles 1–3) as a between-subjects factor. For Retrieval-Status we found a main effect, *F*(2,244) = 231.25, *MSE* = 6.24, *η^2^_p_* = .60, *p*<.001. Post-hoc comparisons indicated improved recall of RP+ (56.2%) and decreased recall of RP− (*M* = 27.5%), compared to NRP items (*M* = 32.1%), *p*<.001. A main effect was found for Set-Type, *F*(1,122) = 146.82, *MSE* = 4.01, *η^2^_p_* = .55, *p*<.001). Post-hoc comparisons indicated that recall for distinct items was reliably higher (*M* = 46.0%) than in the OS (*M* = 31.2%), *p*<.001. Set-Type and Retrieval-Status interacted with each other *F*(2,244) = 8.30, *MSE* = 1.69, *η^2^_p_* = .06, *p* = .001). We have described this interaction in more detail in the next section.

Working Memory Capacity interacted with Retrieval-Status, *F*(4,244) = 3.37, *MSE* = .10, *η^2^_p_* = .05, *p* = .012 and Set-Type, *F*(2,122) = 6.14, *MSE* = .17, *η^2^_p_* = .09, *p* = .003). With regard to Retrieval-Status, post-hoc comparisons indicated that NRP performance was comparable between the three WMC groups (*p*s = .68–1) but high WMC individuals recalled more RP− items than those with low WMC (*p* = .034). With regards to Set-Type, recall of distinct items was comparable for all WMC groups (p≈1) but high WMC individuals recalled significantly more OS items (*M* = 37.3%) compared to both the middle (*M* = 29.0%, *p* = .018) and low group (*M* = 7.3%, *p* = .004). There were no other main effects or interactions of WMC with any other factor (*p*s = .067–1).

### Retrieval induced forgetting and facilitation

To investigate retrieval-induced effects, we used the recall rate of distinct NRP items as the baseline to calculate RIFO and RIFA because it was least affected by interference. For RIFO, we were interested in two separate comparisons to differentiate between within- and between-category effects. Within-category effects, between RP+ and RP− items from the same category were quantified by comparing recall of distinct NRP to the recall of RP− items in both distinct and overlapping sets.. The between-category effect, amongst practiced overlapping categories and related overlapping NRP items, was quantified by comparing performance on distinct NRP and overlapping NRP items. For RIFA effects, distinct NRP was compared to distinct RP+ and overlapping RP+ performance. Figure 1 A and B illustrate the RIFO and RIFA comparisons.

A repeated measures analysis of variance (ANOVA) was conducted with Retrieval-Status (DS NRP, DS RP−, DS RP+, OS NRP, OS RP− and OS RP+) as the within-subject factor and WMC (low, high) as the between subjects factor. A main effect of Retrieval-Status (*F*(5,405) = 87.32, *MSE* = 3.00, *η^2^_p_* = .52, *p*<.001) was observed. Post-hoc comparisons indicated RIFO only for overlapping items, as performance on distinct NRP items was higher than for overlapping NRP and RP− items (*p*<.001). Post-hoc comparisons also indicated RIFA, as distinct RP+ and overlapping RP+ recall was higher than for distinct NRP items (*p*<.01). This effect was qualified by an interaction between Retrieval-Status and WMC (*F*(5,405) = 4.01, *MSE* = .16, *η^2^_p_* = .05, *p* = .001). A post-hoc comparison indicated that contrasted with distinct NRP recall, low WMC individuals had lower recall for overlapping NRP and RP− items, while distinct RP+ recall was higher (ps<.001). The same contrast revealed that high WMC individuals had lower recall of overlapping NRP items, while recall of both distinct RP+ and overlapping RP+ items was higher (ps<.01). See Table 1 for means and Figure 1 for these differences.

Lower performance for overlapping items may partly have been the result of proactive interference. Even though there were always six items per category, participants might have combined the category cue (e.g. sharp or weapon) to form a universal cue (e.g. sharp weapons) which would have led to cue overload and a general decrease of recall for overlapping items; therefore we repeated the analysis only within the OS where interference would have been equal for all items. A repeated measures ANOVA was conducted with Retrieval-Status (OS NRP, OS RP−) as the within-subject factor and WMC (low, high) as the between subjects factor. No main effect for Retrieval-Status was found (*F*(1,81) = 1.25, *MSE* = .03 *η^2^_p_* = .02, *p* = .268) but the Retrieval-Status x WMC interaction approached significance (*F*(1,81) = 3.78, *MSE* = .08 *η^2^_p_* = .04, *p* = .055), indicating a trend toward the result observed in the full, more powerful analysis. A post-hoc comparison indicated that for overlapping items, low WMC individuals' performance on RP− items was significantly lower than for NRP items (*p* = .03) whereas high WMC individuals showed no difference (*p* = .57).

To summarize, we found no RIFO for distinct items. For overlapping items, both low and high WMC individuals exhibited between-category RIFO, while only low WMC individuals exhibited significant within-category RIFO. While proactive interference might have played a role, the pattern of results is consistent within the set in which proactive interference would have been present for all items. RIFA was observed in both groups for distinct items while only high WMC individuals showed better recall of practiced overlapping items.

### Correlation Analysis

To test the relation of WMC and RIFO, we calculated the three RIFO and two RIFA scores per subject and correlated them with the WMC composite score. The correlations are reported in Table 2. In line with our prediction and counter to the earlier findings by Bäuml and Hanslmayer [9], the WMC composite score correlated negatively with the amount of within-category RIFO in the OS. We also found negative correlations between RIFA and RIFO effects, suggesting that individuals who benefitted from retrieval practice failed to report related items.

## Discussion

We have investigated the relationship between WMC and retrieval-induced effects under conditions of high and low interference. Our design included sets of overlapping and distinct items, directly contrasting the effects of low and high interference resolution demands. Factors that are known to influence RIFO, like output interference and integration, were controlled for by using weak items of low taxonomic frequency. In line with the notion that RIFO is caused by resolving interference during retrieval and the subsequent suppression of features [17], we only found RIFO under conditions of high interference. This is in line with the feature suppression model, which states that an item is less likely to be retrieved when its features are inhibited during retrieval practice.

Our findings also support the notion that WMC differences are reflected in retrieval from long-term memory [51], by means of controlled search. Low WMC individuals seemed to enter more irrelevant items into their search set, increasing interference resolution demands, requiring more inhibition, resulting in RIFO within and between overlapping categories. Individuals with high WMC also exhibited between-category RIFO suggesting the effect of some interference but unlike their low capacity counterparts, high WMC individuals showed no RIFO for overlapping items within the practiced category. This is consistent with the idea that high WMC individuals entered fewer irrelevant items into their search set, decreasing interference resolution demands, resulting in no RIFO.

Additionally, in the high interference condition, only high WMC individuals benefited from retrieval practice, which seems to mirror the RIFO effect. Accessibility of an item is determined by the combined effect of retrieval practice, making certain features more accessible, and inhibition, making features less accessible. While executive control is an important component of WMC differences [29] our findings suggest that the ability to delimit the amount of information entered into the search set facilitates retention of practiced information. Only high WMC individuals were able to effectively retrieve similar items from long-term memory during practice and the final memory test, which was evidenced by the negative correlation of WMC and within-category RIFO when interference was high.

The overall negative correlation between WMC and RIFO on the other hand seems to contradict the recent findings of Aslan and Bäuml [9] who reported the opposite result, namely more RIFO for individuals with higher WMC. There are two main differences between our experiments that might explain the disparity: First, the recognition test used by Aslan and Bäuml [9] did not require participants to search their memory but instead to judge familiarity. A recognition test may not require focused search compared to a cued memory test in which retrieving an item based on the correct cue is advantageous. High WMC individuals would arguably benefit from retrieval cues while a recognition test might have greatly aided low WMC individuals, affecting the recall rates for both groups. Second, the delay period between retrieval practice and memory test was considerably shorter, 1 minute compared to 25 minutes. Using longer delays (which are more typical of retrieval-induced forgetting tasks [1]), increases the likelihood that individual differences in efficient retrieval from long-term memory can impact RIFO effects. Individual WMC differences have been argued to manifest themselves in short- and long-term memory [40], [51] but since consolidation takes time, a short delay between retrieval practice and memory test may leave items more active in short-term memory where executive control may play an important role. Both differences may account for the disparity between our findings and those of Aslan and Bäuml [9].

The negative correlation between RIFO and RIFA limits the extent to which we can disregard the effects of blocking. When retrieval of a practiced item prevents access to related items, one would expect that people who show RIFA should also show RIFO. Since using weak items has been found to diminish output interference [45], it is surprising to find any relationship between RIFA and RIFO for the categories where competition between items was low. However, when the data were split up into extreme groups we observed that under conditions of high interference, low WMC individuals exhibited strong RIFO but no RIFA and high WMC individuals showed no RIFO but intact RIFA. No forgetting of competing information and clear benefits of retrieval practice suggests that for high WMC individuals, the search was limited to more relevant information. To our understanding, the blocking account does not predict this dissociation. Our results therefore fit with earlier studies that found RIFA and RIFO to be largely unrelated [52], [53]: RIFA can occur without RIFO [1], [54], [55] and RIFO can occur without RIFA [14], [56], [57]. The dissociation between RIFA and RIFO has also been supported by neuroimaging studies finding different correlates for RIFA and RIFO [4], [26], [58]. Thus, while we cannot exclude the possibility of output interference playing a role, the overall pattern of results fits well with the notion that inhibitory control was used to resolve competition between information in the search set.

When the inhibitory explanation is considered in conjunction with the focused search hypothesis, one may explain why RIFO is found in populations believed to have low executive control like young children [19], [20], people with schizophrenia [21] or Alzheimer's disease [22]. Free recall is often not done in a semantically-clustered fashion for people with schizophrenia [59], children [60] and people with Alzheimer's disease [61], which suggests that their search set is not effectively limited by specific retrieval cues. Within such populations, the effect of committing irrelevant items into the search set might amplify the effect of even low executive control, leading to the observed RIFO effects. While it is essential to control for factors such as integration [24] and output interference [62], we stress that it is also important to consider focused search as a prerequisite for any executive control processes to have an effect.

To summarize, our findings lend support to the inhibitory account of RIFO [1], [25] and the feature suppression model [17]. High WMC individuals seem better able to control interfering information during retrieval from long-term memory which supports the controlled search hypothesis [40], [51] and adds an important dimension to our understanding of retrieval-induced effects which may explain some disparities in the literature. Knowledge about the contribution of controlled search and executive control in high and low interference contexts could be used to inspire new methods of training, especially for people with low WMC who, in our experiment, showed the biggest benefit for remembering items with little feature overlap. Likewise, teaching individuals to use appropriate retrieval cues in certain contexts may be explored.

## Supporting Information

Figure S1 **Retrieval induced effects for high and low working memory capacity individuals.** (A) RIFO scores were calculated by subtracting average performance of DS RP−, OS NRP and OS RP− from DS NRP performance. (B) RIFA scores were calculated by subtracting average performance of DS NRP from OS RP+ and DS RP+ performance. The * and NS show the results of the comparison between DS NRP and respective retrieval status performance. * means the difference is significant, whereas NS means the difference is nonsignificant *p*<.05. In the overlap set, within category, low WMC individuals show RIFO but no RIFA and high WMC individuals show no RIFO but intact RIFA.
(TIF)

Table S1 **Percentage correctly recalled items per condition with standard deviations.**
(DOCX)

Table S2 **Raw Pearson 2-tailed correlations between RIFO and WMC scores (N = 125).** * Significant value *p*<.05.
(DOCX)

Appendix S1 **Words used in the experiment.**
(DOCX)
