# Correction: High Working Memory Capacity Predicts Less Retrieval Induced Forgetting

**DOI:** 10.1371/annotation/feb16b48-f16d-442d-a520-b42aa1b4eb2a

**Published:** 2013-09-19

**Authors:** Jonathan T. Mall, Candice C. Morey

Figure S1 and Tables S1 and S2 were intended to be placed in the main manuscript, not in Supporting Information. The files can be accessed from the electronic version of the article. 

