# Supplementary figures and images for: High Working Memory Capacity Predicts Less Retrieval Induced Forgetting
Source: PLoS One. 2013 Jan 11;8(1):e52806. doi: 10.1371/journal.pone.0052806 (PMC3543406; doi:10.1371/journal.pone.0052806)

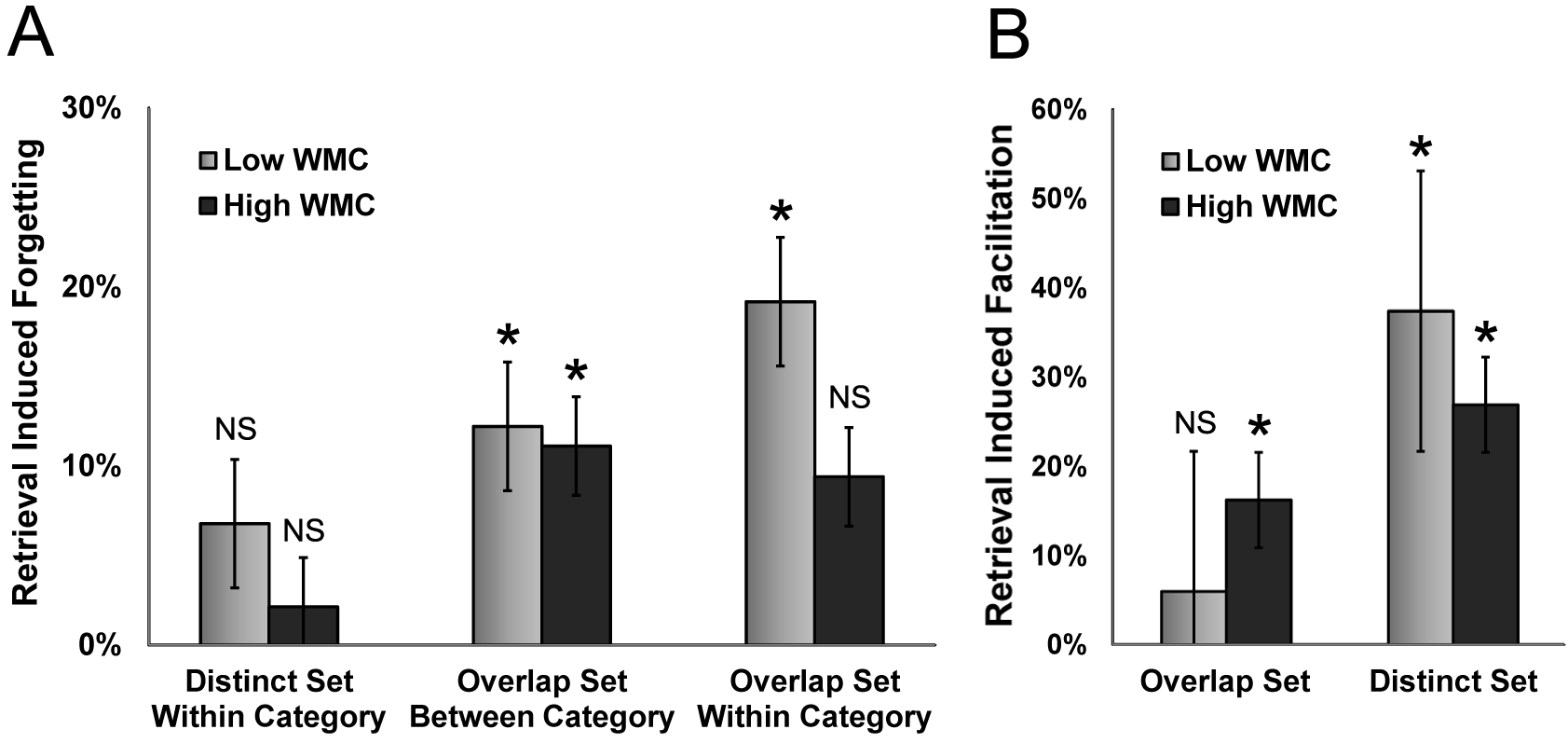

Supplement: Figure S1 — Retrieval induced effects for high and low working memory capacity individuals. (A) RIFO scores were calculated by subtracting average performance of DS RP−, OS NRP and OS RP− from DS NRP performance. (B) RIFA scores were calculated by subtracting average performance of DS NRP from OS RP+ and DS RP+ performance. The * and NS show the results of the comparison between DS NRP and respective retrieval status performance. * means the difference is significant, whereas NS means the difference is nonsignificant p<.05. In the overlap set, within category, low WMC individuals show RIFO but no RIFA and high WMC individuals show no RIFO but intact RIFA. (TIF) [file pone.0052806.s001.tif]
