# Supplementary material for: High Working Memory Capacity Predicts Less Retrieval Induced Forgetting
Source: PLoS One. 2013 Jan 11;8(1):e52806. doi: 10.1371/journal.pone.0052806 (PMC3543406; doi:10.1371/journal.pone.0052806)
Supplement: Table S1 — Percentage correctly recalled items per condition with standard deviations. (DOCX) [file pone.0052806.s002.docx]

**Table 1. Percentage correctly recalled items per condition with standard deviations.**

|  | **Distinct Set** | | | **Overlap Set** | | |
| --- | --- | --- | --- | --- | --- | --- |
|  |  |  |  |  |  |  |
| **Group** | **NRP** | **RP-** | **RP+** | **NRP** | **RP-** | **RP+** |
| Low WMC (N=43) | 35.85(.03) | 29.07(.04) | 73.26(.04) | 23.64(.02) | 16.67(.03) | 41.86(.04) |
| High WMC (N=40) | 38.34(.03) | 35.84(.03) | 64.99(.03) | 27.3(.02) | 29.18(.03) | 55.42(.03) |
