# Supplementary material for: High Working Memory Capacity Predicts Less Retrieval Induced Forgetting
Source: PLoS One. 2013 Jan 11;8(1):e52806. doi: 10.1371/journal.pone.0052806 (PMC3543406; doi:10.1371/journal.pone.0052806)
Supplement: Table S2 — Raw Pearson 2-tailed correlations between RIFO and WMC scores (N = 125). * Significant value p<.05. (DOCX) [file pone.0052806.s003.docx]

**Table S2. Raw Pearson 2-tailed correlations between RIFO and WMC scores (N=125).**

* Significant value *p* < .05.

| **Measure** | **1** | **2** | **3** | **4** | **5** |
| --- | --- | --- | --- | --- | --- |
| 1. Composite WMC Score |  |  |  |  |  |
| 2. Distinct Set Within-Category RIFO | -.05 |  |  |  |  |
| 3. Overlap Set Between-Category RIFO | -.11 | **.24*** |  |  |  |
| 4. Overlap Set Within-Category RIFO | **-.21*** | .08 | **.28*** |  |  |
| 5. Distinct Set RIFA | -.11 | **-.25*** | **-.22*** | **-.35*** |  |
| 6. Overlap Set RIFA | .13 | **-.33*** | **-.32*** | **-.27*** | **.24*** |
