# Supplementary material for: High Working Memory Capacity Predicts Less Retrieval Induced Forgetting
Source: PLoS One. 2013 Jan 11;8(1):e52806. doi: 10.1371/journal.pone.0052806 (PMC3543406; doi:10.1371/journal.pone.0052806)
Supplement: Appendix S1 — Words used in the experiment. (DOCX) [file pone.0052806.s004.docx]

## Appendix S1:

| **Overlap Set:** |  | |
| --- | --- | --- |
| OS items were randomly assigned to create categories with six items for each subject. | | |
| **SCHERPE, WAPENS** | | **VLIEGENDE, DIEREN** |
| degen | | Albaros |
| floret | | Buizerd |
| glas | | flamingo |
| hakmes | | gaai |
| kris | | havik |
| machete | | kip |
| naald | | libel |
| pen | | mees |
| spies | | reiger |
| vork | | specht |
| werpster | | uil |
| zaag | | valk |
| **Distinct Set:** | |  |
| **HOBBY** | | **ZACHT** |
| toneel | | badjas |
| dans | | cavia |
| gitaar | | gras |
| poolen | | poef |
| rugby | | spons |
| surfen | | zeep |
| **KOUD** | | **VOEDSEL** |
| airco | | curry |
| grond | | erwt |
| herfst | | honing |
| iglo | | mais |
| nacht | | salade |
| vorst | | vla |
| **Filler:** | |  |
| **LUID** | | **ZWEMMEN** |
| applaus | | duiker |
| bus | | haring |
| hoorn | | inktvis |
| kermis | | kwal |
| straat | | tonijn |
| zingen | | zalm |
